# Hybrid Solution for Mycotic Pseudoaneurysm of Carotid Bifurcation

**DOI:** 10.1155/2020/8815524

**Published:** 2020-10-19

**Authors:** Giuseppe Deiana, Antonio Baule, Genadi Genadiev Georgiev, Mario Moro, Francesco Spanu, Flavia Urru, Stefano Camparini

**Affiliations:** ^1^Department of Vascular Surgery, Brotzu Hospital, Cagliari, Italy; ^2^Stroke Unit, Public Hospital Santissima Annunziata, Sassari, Italy

## Abstract

Mycotic pseudoaneurysms of the extracranial carotid artery are rare and need surgical treatment to prevent rupture or embolization. We treated a case of a carotid bifurcation pseudoaneurysm secondary to infection caused by *Staphylococcus epidermidis*. We successfully treated it using a catheter balloon to obtain carotid bifurcation's control and replacing the carotid bifurcation with a vein graft. Management involves aneurysmectomy associated with antibiotic therapy and restoration of arterial continuity.

## 1. Introduction

Aneurysms of the extracranial carotid artery are rare [1]. The most frequent cases are secondary to atherosclerosis, trauma, or prior endarterectomy [2–4]. The infected aneurysms of the extracranial carotid artery are even more uncommon, and just few cases have been reported in literature [5, 6].

These aneurysms are associated with high mortality and morbidity [7], also with complications as rupture and metastatic brain abscesses [5]. The management remains a challenge. The use of endovascular stents in infected aneurysms is controversial, and long-term efficacy has not been fully elucidated [8]. We reported a case of mycotic pseudoaneurysm of carotid bifurcation successfully treated with endovascular technique for bleeding control followed by open surgery. Carotid bifurcation has been replaced using a vein graft.

## 2. Case Report

A 54-year-old Caucasian male was admitted to Emergency Care for right cervical swelling present for 1 month and dysphonia appearing in the last week. He suffered from hypertension, ex-smoker addiction, and chronic ischemic heart disease. He denied neck or oral surgery. Vitals were stable, temperature of 38.8°C was reported, and he was hemodynamically stable. On physical examination, there was a pulsatile and hard painless mass in the right anterior neck just above the clavicle, covered by reddened skin. No focal signs of central origin were found.

**Figure 1:**
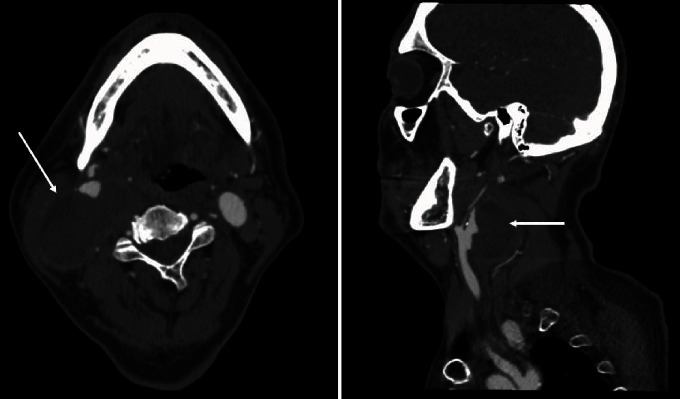
Computed Tomography (CT) scan shows a 60 × 10^5^ mm pseudoaneurysm of the right carotid bifurcation (arrows).

The blood exams revealed an elevated white cell count of 20.96 × 10^3^/*μ*L. Computed Tomography (CT) scan showed a 60 × 10^5^ mm pseudoaneurysm of the right carotid bifurcation surrounded by infected fluid collection (Figure 1) and did not reveal any cerebral embolic signs. We performed echocardiogram and transesophageal echocardiogram negative for endocarditis. Based on clinical and imaging finding, our diagnosis was a mycotic pseudoaneurysm. According to our Infectiology Consultant, the patient started immediately antibiotic therapy with imipenem 500 mg 4 times/day and teicoplanin 400 mg 2 times/day; he received a prophylactic dose of low-molecular-weight heparin during the hospital stay. After 2 weeks of antibiotic therapy, we observed a significative reduction of the mass and we decided to perform surgery.

**Figure 2:**
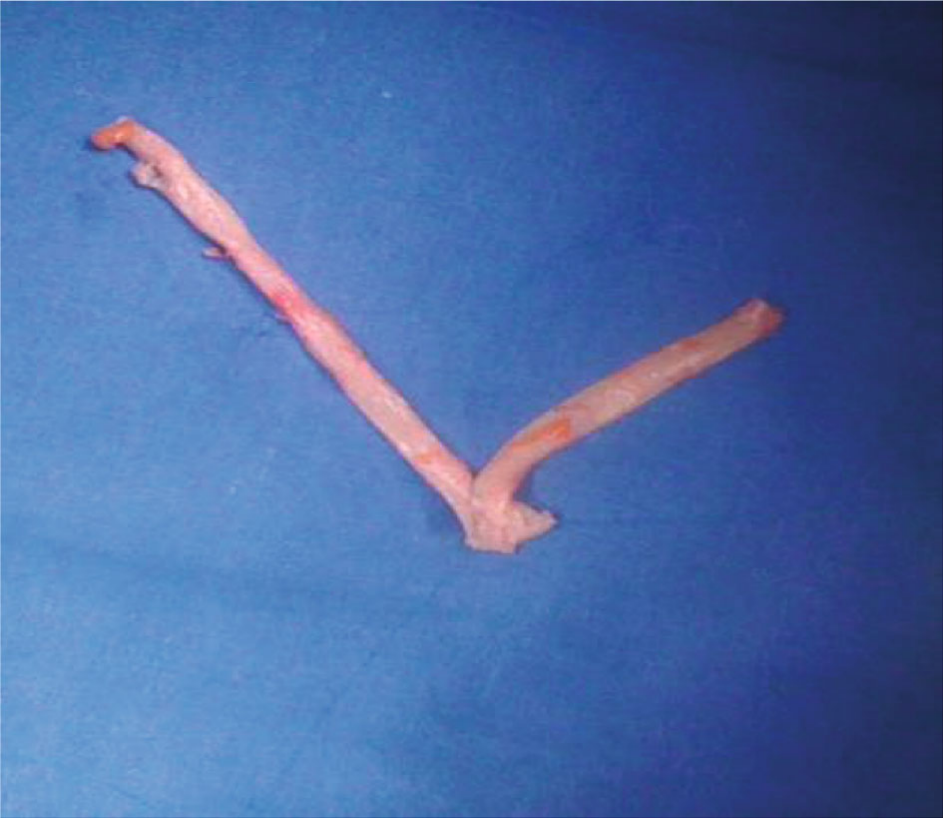
Great saphenous vein prepared to replace the carotid bifurcation.

**Figure 3:**
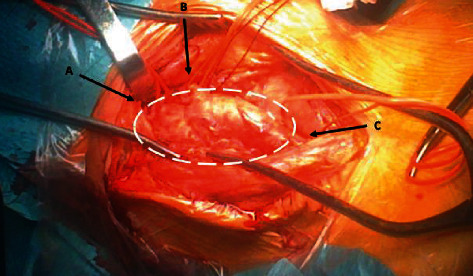
Pseudoaneurysm of right carotid bifurcation after dissection (dashed line) and the control of internal (arrow a), external (arrow b), and commune (arrow c) carotid arteries with vessel loops.

The procedure was performed in general anesthesia, and cerebral perfusion was detected by Near-Infrared Spectroscopy (NIRS). We introduced through the femoral artery a catheter balloon 5 mm for bleeding control of carotid bifurcation. We reconstructed the carotid bifurcation using the vein graft already prepared. Before surgery, we studied the caliber of veins in the upper and lower limbs using ultrasound. We found a satisfactory left great saphenous vein in the right thigh. The saphenous vein was harvested and reversed. We recreated a carotid bifurcation using three segments of the vein. The vessels are being sutured end to end with the parachute technique using Prolene® 6/0 diameters (Figure 2). Classic incision of anterior sternocleidomastoid muscle was performed. We interrupted the blood circulation using the catheter balloon just for the time of dissection of the mass and to achieve the control of internal, external, and commune carotid arteries (Figure 3). After that, the balloon has been retracted clamping, respectively, internal, external, and commune carotid arteries. The pseudoaneurysm sac was opened, and debridement of inflammatory tissue was performed. A sample of the pseudoaneurysm wall was sent for microbiological examination.

**Figure 4:**
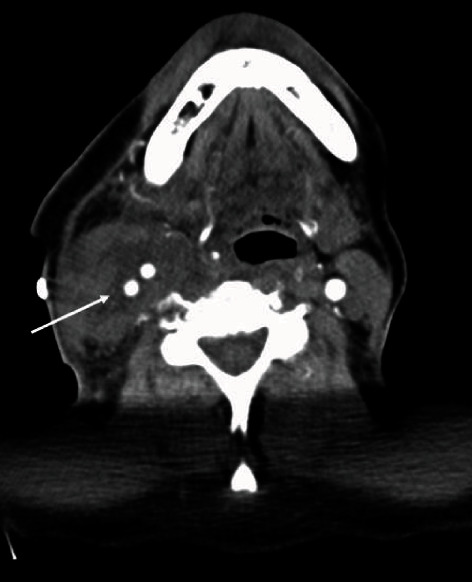
After 1 month from surgery, Computed Tomography (CT) scan shows good patency of the graft (arrow).

At the end of the procedure, angiography showed the regular patency of the graft. The resected tissue grew *Staphylococcus epidermidis*. The postoperation was regular without any central neurological complications. The patient had a normalization of white cell count and all inflammatory indexes; he was discharged after 7 days from surgery, and he continued antibiotic therapy (amoxicillin/clavulanic acid 875 mg/125 mg 2 times/day) for another 2 weeks and aspirin 100 mg once a day. After 1 month, the patient performed a control CT scan that revealed the good patency of the graft (Figure 4) without imaging of cerebral ischemia; moreover, dysphonia recovered completely. Informed consent has been obtained from the patient for publication of the case report and accompanying images.

## 3. Discussion

Mycotic extracranial carotid pseudoaneurysms are rare but can occur due to local or systemic infective processes, like dental suppuration, bacterial sinusitis, bacterial endocarditis, and bacteremia [8]. They represent less than 5% of all arterial pseudoaneurysms [9]. The most common cause of mycotic aneurysms is trauma (42%), but in 25%, the exact source of infection is unknown [10]. In our case, the source remained unclear; the patient was in good general condition. The most common bacterial pathogens associated with mycotic carotid aneurysms are *Staphylococcus*, *Streptococcus*, and *Salmonella* [5]. The same organisms are responsible for the majority of mycotic aneurysms in all anatomic locations [11].

Nowadays, the treatment of choice consists of open surgery and antibiotic therapy.

Usually, the surgical management includes aneurysmectomy, debridement, and restoration of the arterial continuity. Open surgery in the acute setting is generally associated with poor outcomes, including stroke and mortality of up to 50% of patients if the carotid artery is ligated [7]. Due to the risk of septic emboli and rupture during surgery, we preferred to treat the patient with antibiotic therapy before performing the procedure until a reduction of mass volume in the neck was observed. Surgical repair to carotid reconstruction includes the use of autologous arteries, veins, synthetic prosthesis, or cryopreserved arterial allograft. The saphenous vein is considered the first choice because of its resistance to infection and large availability [5]. Some cases of a synthetic prosthesis, associated with medical management, in selected patients demonstrated acceptable outcomes [12]. The synthetic prosthesis is often used in emergency situations when there is not enough time to harvest a vein graft and cryopreserved arterial allograft is not available [13]. Carotid ligation is limited if reconstruction is technically impossible [5]. Endovascular repair has been described as a “bridge” solution before early definitive surgical management [14, 15] or in high-risk patients who are not candidates for open surgery [16]. The use of the balloon could be a two-edged sword, and it could cause embolization, dissection, and bleeding; however, we decided to use the balloon approach due to the mass dimension and to ensure better surgical control of carotid bifurcation. Antibiotic therapy is recommended for at least 6 weeks [7–17], and some authors suggest 6 months [18].

## 4. Conclusions

Mycotic carotid aneurysms are rare. Often, the exact source of infection is unknown. Management involves aneurysmectomy associated with antibiotic therapy and restoration of arterial continuity. Vein graft is to be preferred when possible. The use of a stent graft as a definitive solution is still controversial.

## Data Availability

The authors declared no potential conflicts of interest with respect to the research, authorship, and/or publication of this article.
